# Drugs modulating stochastic gene expression affect the erythroid differentiation process

**DOI:** 10.1371/journal.pone.0225166

**Published:** 2019-11-21

**Authors:** Anissa Guillemin, Ronan Duchesne, Fabien Crauste, Sandrine Gonin-Giraud, Olivier Gandrillon

**Affiliations:** 1 Laboratoire de biologie et modélisation de la cellule. LBMC - Ecole Normale Supérieure - Lyon, Université Claude Bernard Lyon 1, Centre National de la Recherche Scientifique: UMR5239, Institut National de la Santé et de la Recherche Médicale: U1210 - Ecole Normale Supérieure de Lyon 46 allée d’Italie 69007 Lyon, France; 2 Inria Dracula, Villeurbanne, France; 3 Univ. Bordeaux, CNRS, Bordeaux INP, IMB, UMR 5251, F-33400, Talence, France; Brunel University, UNITED KINGDOM

## Abstract

To better understand the mechanisms behind cells decision-making to differentiate, we assessed the influence of stochastic gene expression (SGE) modulation on the erythroid differentiation process. It has been suggested that stochastic gene expression has a role in cell fate decision-making which is revealed by single-cell analyses but studies dedicated to demonstrate the consistency of this link are still lacking. Recent observations showed that SGE significantly increased during differentiation and a few showed that an increase of the level of SGE is accompanied by an increase in the differentiation process. However, a consistent relation in both increasing and decreasing directions has never been shown in the same cellular system. Such demonstration would require to be able to experimentally manipulate simultaneously the level of SGE and cell differentiation in order to observe if cell behavior matches with the current theory. We identified three drugs that modulate SGE in primary erythroid progenitor cells. Both Artemisinin and Indomethacin decreased SGE and reduced the amount of differentiated cells. On the contrary, a third component called MB-3 simultaneously increased the level of SGE and the amount of differentiated cells. We then used a dynamical modelling approach which confirmed that differentiation rates were indeed affected by the drug treatment. Using single-cell analysis and modeling tools, we provide experimental evidence that, in a physiologically relevant cellular system, SGE is linked to differentiation.

## Introduction

Cell-to-cell variability is intrinsic to all living forms, from prokaryotes [1, 2] to eukaryotes [3]. Such variability originates from many sources, but arguably stochastic gene expression (SGE) is an important driving force in the generation of cell-to-cell variability among genetically identical cells [4], although additional regulation layers do exist [5]. Classically, SGE is separated into intrinsic and extrinsic sources [6–10] even if in many cases distinguishing between the two is difficult.

The very existence of SGE led to the concept of a probabilistic mapping between inputs (environment) and outputs (cell decisions) [11]. It is therefore clear that SGE has to be precisely tuned so as to tailor the biological process in which it is involved [12, 13].

As an inevitable consequence of the molecular nature of gene expression process, it is clear that mechanisms dedicated to its control must exist. For example, it has been discussed that during the development, to ensure the reliable reproduction of some particular patterns, cell-signaling devices (*e.g*. the Wnt pathway) act as a noise-filter [14].

By contrast, numerous arguments suggest that SGE variation plays an important role in a wide range of biological processes ranging from bet hedging [15] to the fractional killing of cancer cells [16]. The involvement of SGE in decision-making has been shown in viruses [17–19], in prokaryotes [20–22] and, more recently, in metazoan cells [22–24]. Evidences of its role in the differentiation ability began to emerge from literature through different points of view.

First, the theoretical arguments [25–27], based on evidence of cell-to-cell heterogeneity but without any experimental demonstration, represent differentiation seen from a dynamical point of view [28]: undifferentiated cells exhibit a stable gene expression pattern corresponding to a pre-existing self-renewal state. Once differentiation is activated through external factors, cells could increase their SGE, explore a broader region of their state space and exhibit a mixed gene expression identity between the original and the destination states. Such an exploratory behaviour would increase the probability for cells to attain the space region where they stabilize their new gene expression pattern by reaching another pre-existing stable state: the differentiation state [26, 27]. In other words, an increase of SGE would lead to an improvement of the differentiation dynamic [26]. In addition, it has been described that, in a transition state, the original state may be removed as the gene expression values change, making the process irreversible [27, 29, 30]. Collectively, it has been largely discussed that stochastic fluctuations, associated with extrinsic inputs, appear to be the main means by which cells can change their state [22, 27, 30]. However, this theoretical point of view needs to be supported by biological observations.

Secondly, observation of the role of SGE during differentiation emerged, some years later, through experimental measures of the amount of SGE during differentiation processes [31–34]. We recently described a surge in cell-to-cell variability that precedes an irreversible differentiation of normal primary chicken erythroid progenitors called T2EC [35], that is fully compatible with such a view [31]. Interestingly, these results have been confirmed in various settings, ranging from the differentiation of murine lymphohematopoietic progenitors [33] to the differentiation of murine embryonic stem cells [32, 34].

Finally, experimental demonstrations such as studies of artificial modulation of the amount of SGE [13, 36] started to emerge. These last types of evidences aim at reinforcing the consistency of the relation between SGE and differentiation but a clear demonstration and characterization of this link is still lacking. Here, we pave the way toward this demonstration, adding a new complementary study to the legacy.

To do so, it is necessary to show that pharmacological modulators such as drugs [12, 13, 36] would on one hand modify SGE and, on the other hand modulate the differentiation process. It has recently been described that such drugs, identified using a large screening approach, were able to modulate the noise affecting a LTR-driven reporter gene in human T-lymphocytes [37]. In addition, drugs that directly inhibit promoter nucleosome remodelling were also shown to provide fine-tuning of SGE [38].

In order to demonstrate the general aspect of the relation between SGE and differentiation, we decided to explore the extent to which some of those drugs, that are able to reduce (Artemisinin and Indomethacin) or to increase (MB-3, [7]) the level of SGE, could alter differentiation.

Here we show that the three selected drugs modify, significantly and simultaneously, the level of SGE and the rate of cell differentiation. We therefore provide a clear evidence that, in a physiologically relevant cellular system, a pharmacological modulation of SGE is positively and consistently accompanied by a modification of differentiation, as suggested by existing points of view.

## Materials and methods

### Cell culture and treatment

T2EC were extracted from the bone marrow of 19 days-old SPAFAS white leghorn chickens embryos (INRA, Tours, France). These cells were maintained in a medium called LM1. It is composed of *α*-MEM medium supplemented with 10% Foetal bovine serum (FBS), 1 mM HEPES, 100 nM *β*-mercaptoethanol, 100 U/mL penicillin and streptomycin, 5 ng/mL TGF-*α*, 1 ng/mL TGF-*β* and 1 mM dexamethasone as previously described [35]. T2EC were induced to differentiate by removing the LM1 medium and placing cells into the DM17 medium (*α*-MEM, 10% foetal bovine serum (FBS), 1 mM Hepes, 100 nM *β*-mercaptoethanol, 100 U/mL penicillin and streptomycin, 10 ng/mL insulin and 5% anemic chicken serum (ACS)). Differentiation kinetics were obtained by collecting cells at different times after the induction in differentiation. For Indomethacin and Artemisinin, cells in the self-renewing medium are treated at respectively 25 *μ*M and 1 *μ*M 48h before switching into a differentiated medium in order to optimize their effects. For MB-3 ((2R,3S)-rel-4-Methylene-5-oxo-2-propyltetrahydrofuran-3-carboxylic acid), cells are treated at 10 *μ*M just after inducing the differentiation. For each drug, a control treatment (0.1% DMSO) was added following the same conditions.

### Counting of cell viability and cell differentiation

Cell population growth was evaluated by counting living cells using a Malassez cell and Trypan blue staining (SIGMA). This method was also used to assess the toxicity of the drugs in T2EC (S6 Fig). Cell population differentiation was evaluated by counting differentiated cells using a counting cell and Benzidin (SIGMA) staining which stains haemoglobin in blue.

### Dynamical model for erythroid differentiation

Every detail regarding the design, selection, calibration or identifiability analysis of our dynamic model can be found in its original paper [39]. All data and pieces of code used for the curent study are available in a public github repository https://github.com/rduchesn/Drugs-modulating-stochastic-gene-expression-affect-the-erythroid-differentiation-process. Herein, we give only the definitions of the useful concepts and methods in this study.

#### Model definition

**Dynamic model** The ODE governing the time-evolution of the cell populations in each compartment of the model are given in Eq 1:

$$\frac{dS}{dt}=\rho_{S}S\left(t\right)-\delta_{SC}S\left(t\right),$$ (1a)

$$\frac{dC}{dt}=\rho_{C}C\left(t\right)+\delta_{SC}S\left(t\right)-\delta_{CB}C\left(t\right),$$ (1b)

$$\frac{dB}{dt}=\rho_{B}B\left(t\right)+\delta_{CB}C\left(t\right).$$ (1c)

It is characterized by the set (*ρ*_*S*_, *δ*_*SC*_, *ρ*_*C*_, *δ*_*CB*_, *ρ*_*B*_) of five parameters, where *ρ*_*i*_ is the net proliferation rate of compartment *i* (positive or negative), and *δ*_*ij*_ is the differentiation rate of cell type *i* into cell type *j*, which is positive.

The solution to this linear ODE model is given in the supplementary materials of the original paper [39]. We will note *f*_*i*_(*t*_*j*_, *y*_0_, *θ*) the prediction of this model for the *i*^th^ observable of the experiment, on the *j*^th^ timepoint *t*_*j*_, with the initial condition *y*_0_ and parameters *θ*.

**Error model** In order to properly define the statistical likelihood of our model, we introduce a gaussian model for the distribution of the residuals of the dynamic model:

$$\begin{array}{c} y_{i,j,k}\;↪\;N(f_{i}\left(t_{j},y_{0},\theta \right),b_{i}.f_{i}\left(t_{j},y_{0},\theta \right)), \end{array}$$ (2)

where *y*_*i*,*j*,*k*_ is the experimental measure for the *i*^th^ observable, at the *j*^th^ timepoint, in the *k*^th^ repetition of the experiment. Here *b*_*i*_ is an error parameter which quantifies the variance of the model residuals, and should be estimated together with the parameters *θ* of the dynamic model.

#### Estimation in the control case

**Likelihood** From Eq 2, the likelihood of the model naturally follows, and we can estimate the best-fit parameter values of the model by minimizing the negative logarithm of the likelihood:

$$\begin{array}{c} -2\;log\left(L\right)=\sum_{i=1}^{n}\sum_{j=1}^{m}\sum_{k=1}^{l}{\left(\frac{\left(y_{i,j,k}-f_{i}\left(t_{j},\theta \right)\right)}{b_{i}.f_{i}\left(t_{j},y_{0},\theta \right)}\right)}^{2}+2\;log\left(b_{i}.f_{i}\left(t_{j},y_{0},\theta \right)\right), \end{array}$$ (3)

where n is the number of observables of the model, m the number of timepoints, and l the number of repetitions of the measurements.

**Algorithmic details** We minimized −2 *log*(*L*) using the Truncated Newton’s algorithm [40, 41] implemented in the python package for scientific computing scipy [42]. Convergence to the global minimum was assured by a random sampling of the initial guesses for parameter values.

#### Estimation under treatment

**Parameter variations** The model has seven parameters (five dynamic parameters: *ρ*_*S*_, *δ*_*SC*_, *ρ*_*C*_, *δ*_*CB*_ and *ρ*_*B*_; and two error parameters: *b*_1_ and *b*_2_), of which six are estimated from the data. Under a given treatment, we consider that each estimated parameter could either be estimated from the data, or set equal to its control value. For each treatment, this defines 2^6^ = 64 different models, with a varying number of additional parameters to estimate.

**Model selection** We estimated the parameter values of these 64 models for each treatment, and selected the best models by computing their corrected Akaike’s Information Criterion [43]:

$$\begin{array}{c} AICc=-2\;log\left(L\right)+\frac{2kn}{n-k-1}. \end{array}$$ (4)

where *k* is the number of parameters of the model and *n* is the sample size. From the corrected AIC, we compute the Akaike’s weights:

$$w_{i}=\frac{exp({-}^{\left(AICc_{i}-min(AICc)\right)}/2)}{\sum_{j=1}^{R}exp({-}^{\left(AICc_{i}-min(AICc)\right)}/2)},$$ (5)

where *w*_*i*_ is the Akaike’s weight of the i-th model, and *R* = 64 is the number of competing models. The Akaike’s weight of a given model in a given set of models can be seen as the probability that it is the best one among the set [43]. In this setting, selecting the best models of a set of models means computing their Akaike’s weights, sorting them, and keeping only the models whose weights add up to a significance probability (in our case, 95%).

### Single cell high-throughput RTqPCR

Every experiment related to high-throughput microfluidic-based RT-qPCR was performed according to Fluidigm’s protocol (PN 68000088 K1, p.157-172) and recommendations. All the following steps from single-cell isolation to high throughput RTqPCR of each cells are described in [31].

### Entropy

We estimated the Shannon entropy of each gene *j* at each timepoint *t* as follows: we computed basic histograms of the genes with N = Nc /2 bins, where Nc is fixed for all tests, which provided the probabilities $p_{j,k}^{t}$ of each class k. Finally, the entropies were defined by

$$\begin{array}{c} E_{j}^{t}=-\sum_{k=1}^{N}p_{j,k}^{t}\log_{2}\left(p_{j,k}^{t}\right). \end{array}$$

When all cells express the same amount of a given gene, this gene’s entropy will be null. On the contrary, the maximum value of entropy will result from the most variable cell-to-cell gene expression level.

## Results

### Drugs affect noise

In order to characterize the relation between SGE and differentiation, we first make sure to be able to change the amount of SGE in T2EC using three drug treatments: Artemisinin, Indomethacin and MB-3.

Artemisinin and Indomethacin are known to modify SGE of the HIV LTR promoter in human T-lymphocytes [37]. MB-3, a chromatin modifier, is known to modify stochastic gene expression in yeast [7] and in murine embryonic stem cells [13]. At first, we wanted to confirm that these drugs do indeed modify SGE in our cellular system and to determine the mechanisms associated with this effect.

We treated T2EC with or without drugs and induced their erythroid differentiation. We then performed single-cell high-throughput RTqPCR on these cells at different time points after differentiation. We assessed a 92 gene panel, relevant for erythroid differentiation study, identical to those previously measured in untreated cells [31].

There are various ways of quantifying the amount of so-called “noise” in gene expression. Unfortunately, no consensus has emerged with authors advocating for the use of normalized variance (*NV* = *σ*^2^/*μ*^2^) and claiming that the use of Fano Factor (*F* = *σ*^2^/*μ*) might be misleading [44, 45], whereas others defend the exact opposite position [46]. The use of the coefficient of variation (*CV* = *σ*/*μ*) is also known to be limited [45, 47–49].

Both we [31] and others [32, 50, 51] have recently proposed the shannon entropy as an alternative measure, that is dedicated to quantify cell-to-cell variability. For our purpose and based on its mathematical definition, a deterministic pattern of expression exhibits low entropy whereas a high entropy indicates a more diverse expression pattern [31, 50]. We therefore analyzed our single-cell transcriptomic data using this metric.

**Fig 1:**
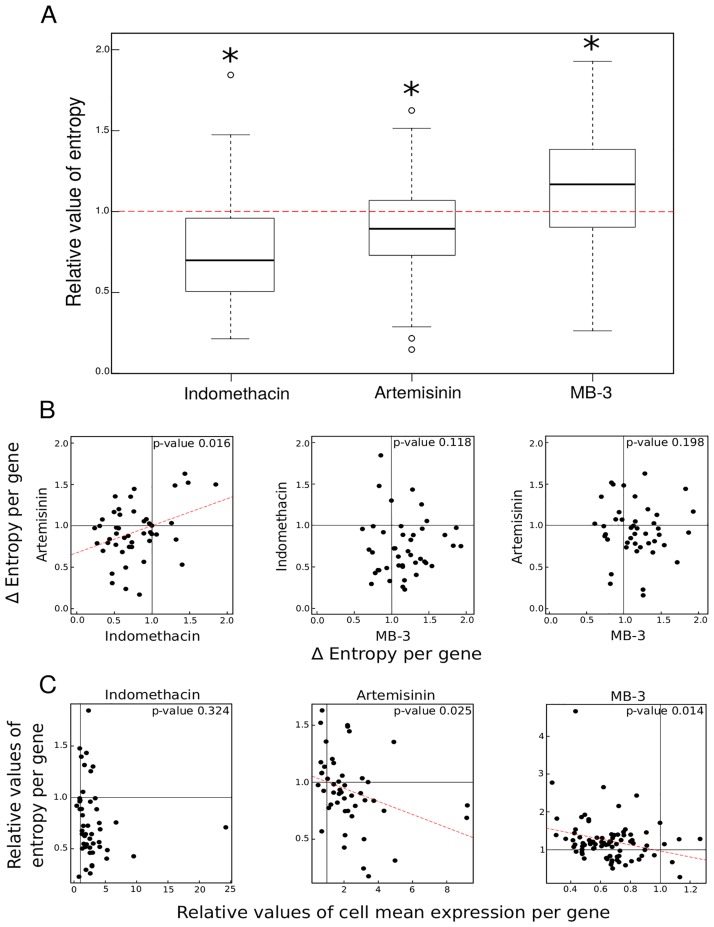
Relative effect of entropy and average gene expression level under drug treatment during differentiation. (A) Boxplots representing values of entropy per gene for each treatment relative to control values (red dotted line). Some outliers are not displayed for readability. We assessed the significance of the differences between untreated and treated conditions through a Wilcoxon test (tests with a p-value < 0.05 are represented by a star above each boxplot). (B) Correlation plots representing relative values of entropy per gene for each pair of drugs. We assessed the significance of the differences between values for each drug through a Pearson test (p-value < 0.05). When the correlation is significant, we displayed the linear regression line for all points (red dotted lines). (C) Correlation plots representing relative values of entropy as a function of relative values of cell mean expression per gene. We assessed the significance of the differences between values for each drug through a Pearson test (p-value < 0.05). When the correlation is significant, we displayed the linear regression line for all points (red dotted lines).

We can observe in (Fig 1A) that the entropy was affected by all treatments. Under Indomethacin or Artemisinin treatment, entropy significantly decreased after 2 days of erythroid differentiation. This effect was more pronounced with Indomethacin. The opposite effect is observed with the MB-3-based treatment, for which entropy was significantly increased after 12h of differentiation (Fig 1A).

We then assessed whether the same genes exhibit entropy variations under the different drug treatments or not. To do so, we computed a correlation value between the entropy variations for each pair of drugs. If the same genes are affected by two drugs, then one would expect their entropy variations to be correlated. We observed a significant correlation only for the genes affected by Indomethacin and Artemisinin treatment. MB-3 treatment seemed to affect the variability of a different set of genes (Fig 1B).

The entropy variation could be achieved by modulating the global mean gene expression or the gene expression variance. Thus we finally wanted to test if our drug treatments affected entropy through the modulation of the mean gene expression value. If so, one might expect to see a correlation between the variation of entropy and the mean expression level under drug treatment.

Indeed for two drugs out of three, Artemisinin and MB-3, we observed a significant inverse correlation between mean and entropy (Fig 1C). Nevertheless the effect of Indomethacin on entropy was not related to an effect on mean gene expression.

Here we have found three drugs that modulate SGE in T2EC cells. Indomethacin and Artemisinin decreased it whereas MB-3 increased it. MB-3 involved a different set of genes than Artemisinin and Indomethacin (also shown in S1 Fig) and the effect of drugs was not strongly related to an effect on mean gene expression value. Entropy modulation is therefore the only common characteristic of our three drugs.

Even with a weak correlation, it seems that Artemisinin and Indomethacin could affect the same genes to change the level of SGE. Thus, we decided to get more insight on genes targeted by each drugs by selecting genes of interest in function of the influence of drugs on them (Table 1).

**Table 1:** Relative entropy values. Here are displayed the gene entropy values across cells for each treatment normalized by the control condition. Bold values represent entropy ratio strictly superior to the first quartile (q1, top values) or strictly inferior to the third quartile (q3, bottom values). Genes represented are the genes which RTqPCR quality control passed. The time points used to show these values are the same as in Fig 1A. Values are ordered from the highest to the lowest ratio. Genes in bold are those on which we have focused. RE = Relative Entropy.

INDOMETHACIN		ARTEMISININ		MB-3			
Genes	RE	Genes	RE	Genes	RE	Genes	RE
**ALAS1**	**1.8455**	**MFSD2B**	**1.6275**	ACSL6	**4.7113**	WDR91	1.1068
**TPP1**	**1.4769**	**TPP1**	**1.5182**	AMDHD2	**3.1564**	HSD17B7	1.1061
**MFSD2B**	**1.4329**	**ALAS1**	**1.4970**	GPT2	**2.8459**	GSN	1.1055
AMDHD2	**1.3961**	**MKNK2**	**1.4845**	PLAG1	**2.4347**	DPP7	1.0989
GPT2	**1.3151**	CYP51A1	**1.4429**	SQLE	**2.4193**	SULT1E1	1.0959
**MKNK2**	**1.3005**	PLS1	**1.3522**	CYP51A1	**2.2100**	TADA2L	1.0708
EGFR	**1.2545**	CRIP2	**1.3489**	EMB	**1.8670**	PIK3CG	1.0682
FNIP1	**1.0539**	VDAC3	**1.1979**	RPL22L1	**1.8081**	SLC25A37	1.0479
FHL3	**0.9916**	FDFT1	**1.1695**	BCL11A	**1.7895**	BETA-GLOBIN	1.0463
DHCR24	**0.9906**	LCP1	**1.1626**	FDFT1	**1.7042**	STX12	1.0313
EMB	**0.9718**	HSP90AA1	**1.1299**	SLC9A3R2	**1.6493**	NSDHL	1.0148
SMPD1	**0.9609**	PIK3CG	**1.0756**	BATF	**1.6040**	**MKNK2**	0.9976
HMGCS1	**0.9580**	q1		TBC1D7	**1.5405**	RSFR	0.9514
q1		STARD4	1.0726	SMPD1	**1.5150**	LCP1	0.9367
PIK3CG	0.9183	SLC6A9	1.0507	VRK3	**1.4939**	q3	
SLC6A9	0.8871	EGFR	1.0281	UCK1	**1.4604**	PPP1R15B	**0.8977**
SLC9A3R2	0.8849	HMGCS1	1.0235	RFFL	**1.4594**	STARD4	**0.8758**
GLRX5	0.8844	FHL3	0.9984	HSP90AA1	**1.4416**	DHCR24	**0.8593**
RBM38	0.8261	DCTD	0.9938	**HRAS1**	**1.4317**	RUNX2	**0.8558**
CYP51A1	0.7558	SULT1E1	0.9741	TTYH2	**1.4216**	**ALAS1**	**0.8556**
FDFT1	0.7508	SQSTM1	0.9674	MAPK12	**1.4154**	BPI	**0.8393**
BETA-GLOBIN	0.7245	UCK1	0.9656	q1		**TPP1**	**0.8330**
STX12	0.7230	GAB1	0.9256	FNIP1	1.4070	**LDHA**	**0.8306**
CRIP2	0.7086	EMB	0.9168	GLRX5	1.3965	SCA2	**0.8176**
CREG1	0.6893	GLRX5	0.9032	HYAL1	1.3823	CD44	**0.8141**
PDLIM7	0.6750	NCOA4	0.9010	DHCR7	1.3723	SULF2	**0.7960**
REXO2	0.6438	DHCR24	0.8958	**MFSD2B**	1.3401	SCD	**0.7788**
SQLE	0.6427	FNIP1	0.8904	CTSA	1.3288	CRIP2	**0.7530**
GAB1	0.6423	PDLIM7	0.8738	VDAC3	1.3207	HMGCS1	**0.7420**
CTCF	0.6247	CTCF	0.8496	PTPRC	1.2901	DCTD	**0.7298**
**HRAS1**	0.5975	SULF2	0.8332	CD151	1.2687	SERPINI1	**0.7197**
HSP90AA1	0.5646	GPT2	0.8289	RBM38	1.2577	PDLIM7	**0.6980**
VDAC3	0.5511	SMPD1	0.8124	REXO2	1.2559	ACSS1	**0.6950**
UCK1	0.5481	BETA-GLOBIN	0.7950	PLS3	1.2558	SNX22	**0.6351**
RPL22L1	0.5424	VRK3	0.7881	EGFR	1.2491	SNX27	**0.5169**
NCOA4	0.5184	DCP1A	0.7837	MVD	1.2472	FAM208B	**0.2530**
SULT1E1	0.5174	TBC1D7	0.7654	SQSTM1	1.2446		
q3		q3		PLS1	1.2412		
TBC1D7	**0.5109**	CREG1	**0.7399**	AACS	1.2411		
PLS1	**0.5022**	STX12	**0.7391**	CREG1	1.2202		
LCP1	**0.4868**	RPL22L1	**0.7372**	XPNPEP1	1.2014		
SCA2	**0.4622**	**MTFR1**	**0.6919**	SLC6A9	1.1889		
**LDHA**	**0.4602**	**HRAS1**	**0.6778**	ARHGEF2	1.1863		
SULF2	**0.4267**	SLC9A3R2	**0.5585**	FHL3	1.1835		
VRK3	**0.4040**	AMDHD2	**0.5245**	**MTFR1**	1.1724		
**MTFR1**	**0.3391**	SQLE	**0.4894**	DCP1A	1.1485		
STARD4	**0.3304**	**LDHA**	**0.4151**	NCOA4	1.1460		
DCTD	**0.2950**	SCA2	**0.3003**	CTCF	1.1279		
DCP1A	**0.2602**	REXO2	**0.2303**	MID2	1.1165		
SQSTM1	**0.2276**	RBM38	**0.1613**	TNFRSF21	1.1085		

We observed that for some of the genes that we studied, the entropy was affected by both Indomethacin and Artemisinin. We therefore decided to focus our analysis more specifically on these genes.

From the Shannon entropy value of each gene for each condition, we calculated the relative value for each treatment compared to the control and reordered these results from the highest ratio to the weakest. For a given gene in a given treatment, if this relative entropy value is very different from one, it means that the entropy of that gene was greatly affected by the treatment. In such a way, genes are represented in function of their entropy ratio values from top to bottom as the most positively affected by drugs to the most negatively affected (Table 1). The gene expression distributions of the most affected genes (negatively and positively) for each drugs were represented in S2 Fig. The first quartile of the resulting distribution of relative entropy indicates genes for the entropy was the most positively affected by the treatment. Conversely, the third quartile indicates genes for which the entropy was the most negatively affected by the treatment.

We can observe that for Indomethacin and Artemisinin treatments, the 3 top genes that are the most positively affected by the drugs in variability are the same: *ALAS1*, *TPP1* and *MFSD2B*. Whereas, for MB-3, the relative entropy of *TPP1* and *ALAS1* genes are negatively affected (under the third quartile). Such as Indomethacin and Artemisnin, MB-3 affected positively the entropy of *MFSD2B*. We can also find the gene *MKNK2* as a common positively affected gene for Indomathacin and Artemisinin (all their relative entropy values are superior to the first quartile). Under MB-3 treatment, the relative entropy of *MKNK2* does not seem to be affected as its value is between the first and the third quartile (Table 1). For the genes that are most negatively affected by drugs, the results are less clear. Under the third quartile, we can find *MTFR1* and *LDHA* for both Artenisinin and Indomethacin. For MB-3, *LDHA* is negatively affected. HRAS1 is also negatively affected by Artemisinin and Indomethacin but its value is not under the third quartile for the Indomethacin treatment. *HRAS1* is positively affected under the MB-3 treatment but for *MTFR1*, the relative entropy value is unchanged (Table 1). Regarding gene expression distributions, the most positively affected genes (in terms of entropy), show wider distribution in the treated condition than in the control (S2 Fig). The inverse is true with the most negatively affected genes. That supports the relevance of the indicator used, Shannon’s entropy. Moreover, all the distributions are heavy-tailed, which is expected from mRNA single cell distributions [52].

As we have shown previously, to modulate the level of SGE, drugs did not target a specific set of genes. This is clearer for MB-3 compared with the two others than between Indomethacin and Artemisinin. However, the increase of the noise level of *ALAS1*, *TPP1* and *MFSD2B* seems to be a common effect of Artemisinin and Indomethacin.

In addition, to support these results, we decided to analyse *in silico* the connections between the three drugs’ targets.

To do so, we compared the different targets known in literature. For MB-3, the only target known is KAT2A protein [7, 13, 53]. Indomethacin targets both Cyclooxygenases (COX-1 and COX-2 also called PTGS for Prostaglandin-Endoperoxide Synthase) [54]. For Artemisinin, the task to find its targets is more complex because of the unspecificity of this drug [55]. In 2019, Heller and Roepe listed targets of Artemisinin-based drugs among three proteomic studies [56].

All together, known connections between proteins were represented using the STRING database (http://string.embl.de/) in (S1 Fig). Each edge between two proteins corresponds to a known association between those proteins. We can observe that KAT2A is alone in the connection network. Both PTGS-1 and PTGS-2 are highly correlated together but poorly correlated with the rest of the network. Each link refers to a co-mention between these two terms in a PubMed Abstract. None of them [57, 58] shows a direct interaction between these molecules. All these results suggest that there is no direct interaction between the drug targets reported in the literature. However, we have to keep in mind that we only compare the data reported in literature and that a potential interaction between drugs remains possible but not yet discovered.

We next used these drugs to test their effect on the erythroid differentiation process.

### Drugs affect differentiation

**Fig 2:**
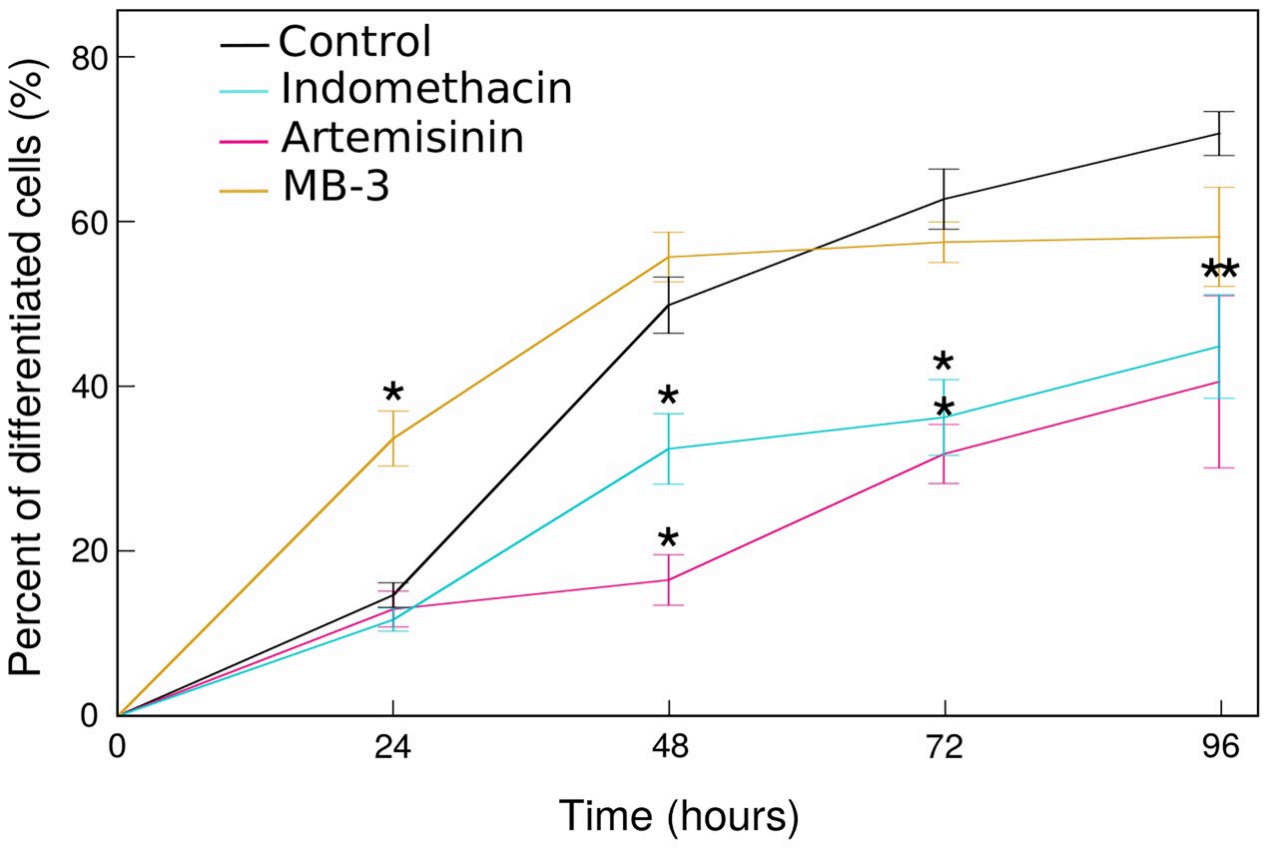
Drugs affect erythroid differentiation. Control conditions were averaged (black line) for readability. Shown is the percentage of differentiated cells for all conditions. Error bars represent the standard-deviation between experiments (n = 3). We assessed the significance of the differences between each treated condition with their own control condition through a student test (p-value < 0.05).

In order to know if drugs modulating SGE also affect the differentiation process, we measured the percentage of differentiated cells in treated and untreated conditions during 96h of erythroid maturation (Fig 2).

A significant modulation in the percentage of differentiated cells was observed for all three drugs (Fig 2).

Indomethacin and Artemisinin decreased the percentage of mature cells from 48h of differentiation onward. MB-3 acted earlier: it significantly increased the percentage of differentiated cells by 24h before returning to somewhat below the control level.

Indomethacin and Artemisinin, two drugs that decreased SGE, reduced the percentage of differentiated cells. Inversely, MB-3 that increased SGE, enhanced the percentage of differentiated cells.

However, at this stage, we cannot conclude that a modification of the level of SGE by drugs is associated with a change of the differentiation process itself. Indeed, these effects might have several origins including modification in growth or death rates of our cells, which we cannot measure experimentally. To decipher between these effects, we decided to use a mathematical model describing the dynamics of the *in vitro* erythroid differentiation [39].

### Cellular basis of drug effect

**Fig 3:**
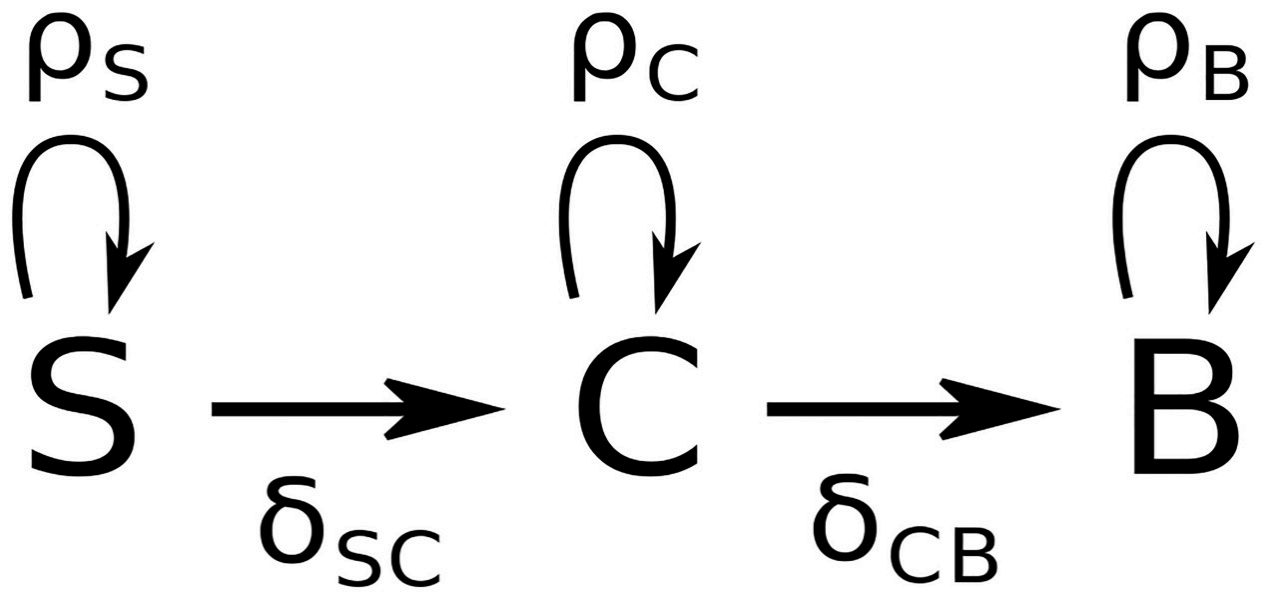
Schematic diagram of the model.

Our model describes the dynamics of three cell populations related to three different stages of differentiation [39]. The first one is the self renewing state (S) where differentiation has not started; the third one is the differentiated state (B) where cells have finished differentiating. The second one is the committed state (C), comprising intermediary cells that are committed to differentiation but not yet fully differentiated (Fig 3). The dynamics of these compartments follow a set of linear ODE. From the size of the cell population in the culture (S3 Fig), it seems reasonable to use a deterministic framework when modelling the growth and differentiation of the whole population.

Our dynamic model is characterized by a set of five parameters *θ* = (*ρ*_*S*_, *δ*_*SC*_, *ρ*_*C*_, *δ*_*CB*_, *ρ*_*B*_):

- *ρ*_*i*_ is the proliferation rate of compartment *i*, involving the balance between cell proliferation and cell death. This value can be either positive (more proliferation than death) or negative (more death than proliferation).
- *δ*_*ij*_ is the differentiation rate of cell type *i* into cell type *j*, which is positive.

Considering that there remains no self-renewing cells after 2 days of T2EC differentiation (S4 Fig, [31]), *δ*_*SC*_ is a fixed parameter fully determined by *ρ*_*S*_ [39].

In order to get the best description of the drugs effects with the fewest parameters, we used the same approach as described in [39] and in the Methods section.

First, we estimate the parameters of the dynamic model in the control condition, using the data presented in Fig 2 (living cells and differentiated cells counts in the self-renewal and the differentiation media). We have already proven that our model identifiable, both theoretically and practically [39], using the profile likelihood approach [59]. It thus makes sense to compare the parameter values between the treated and untreated conditions.

For a given treatment, we consider that each parameter could either be equal to its untreated value, or to another value which should be estimated from the data (thus introducing a new parameter in the model). We test all the combinations of parameters that might vary under each treatment, and we select the best ones using Akaike’s weights, that are displayed on S5 Fig [43].

**Fig 4:**
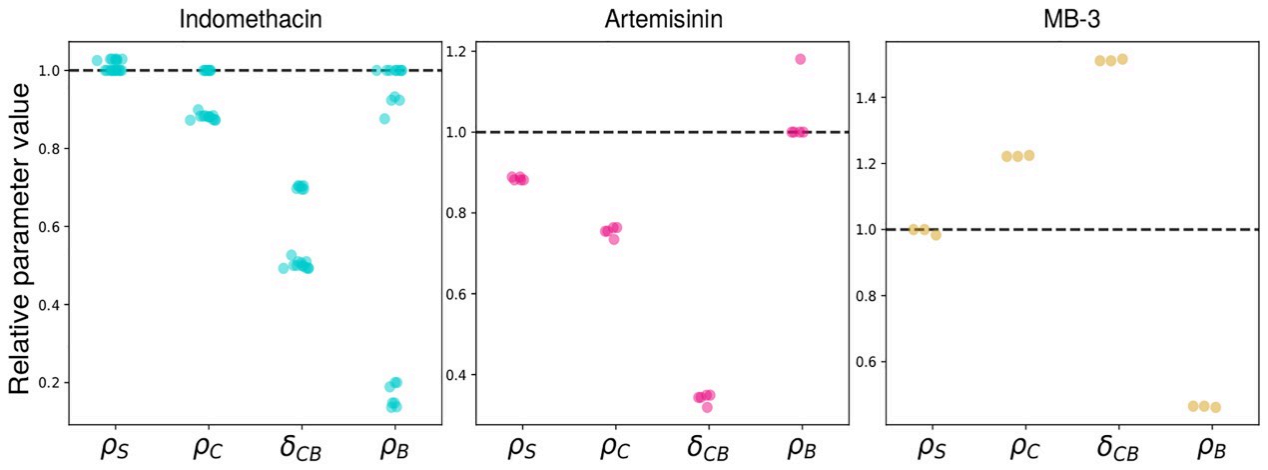
Relative parameter values. For each of the models selected by Akaike’s weights (S5 Fig), all the relative parameter values are represented by a dot for a treatment compared to the untreated condition (black dotted line). Among all the combinations of parameters that might vary under each treatment, 19 models were selected for the Indomethacin treatment using Akaike’s weights, 5 for the Artemisinin treatment and 3 for MB-3. The horizontal spacing between the values of each parameter was chosen randomly for readability.

In the end, the parameter sets that we display in Fig 4 are those that reproduced well, and with the fewest additional parameters, the cellular kinetics during the *in vitro* differentiation (S3 Fig).

For all of those best models, the parameter values for each treatment are displayed in Fig 4. Under Indomethacin or MB-3 treatment, *ρ*_*S*_ (net growth rate of the immature cells) was not affected in all models and slightly decreased under Artemisinin treatment. Therefore, *δ*_*SC*_ was not affected by the treatments either, since its value is entirely determined by the value of *ρ*_*S*_.

Concerning *ρ*_*C*_, the net growth rate of the committed compartment, its values were reduced compared to the untreated condition for the majority of models under Indomethacin or Artemisinin treatment, whereas for MB-3 its value increased in all models.

A more variable change between drug effect was observed with parameter *ρ*_*B*_, which describes the net growth rate of differentiated cells. Under Indomethacin treatment, some of the best models did not show a different value when compared to untreated condition whereas some models displayed a reduced parameter value. Under Artemisinin treatment this value was unchanged for four models among five and increased for the other one. With MB-3 treatment, *ρ*_*B*_ decreased in all models.

Finally, we found that the *δ*_*CB*_ parameter, representing the differentiation rate between committed compartment and mature cell compartment was affected by all three drugs: both Indomethacin and Artemisinin reduced this differentiation rate whereas MB-3 increased it in all best models.

These results demonstrate that all three drugs alter the differentiation process by modifying all dynamical parameters including the differentiation rate between committed and mature cells. It is clear that drugs that reduce SGE decrease the differentiation rate T2EC and inversely that the drugs increasing SGE accelerate cell differentiation, in line with our initial hypothesis.

## Discussion

In this study, we highlighted for the first time the existence of a relation between the pharmacological modulation of stochastic gene expression and differentiation in both directions (increase or decrease) in the same cellular system. We first showed that three drugs, selected from literature [7, 37], increase or decrease the level of SGE in our cells. We therefore tested their effect on the differentiation ability of avian erythropoietic progenitors. We identified which differentiation parameters were affected by drugs using a dynamical model of the *in vitro* erythroid differentiation [39]. We demonstrated that the modulation of the differentiation process impacted the differentiation rate between the last two compartments. We therefore demonstrated that drugs modulating the amount of SGE simultaneously modify the differentiation process supporting all existing points of view [22, 25–27, 30] and reaching toward recent experimental evidences [13, 36].

Indomethacin, Artemisinin and MB-3 have clearly different functions. Artemisinin is an antimalarial drug used against a parasitic infection [53]. Indomethacin is an anti-inflammatory drug that affects the prostaglandin pathway [54]. These drugs were selected from another study [37] for their effect on the level of SGE on a HIV LTR promotor in human lymphocyte line. In this study, Artemisinin and Indomethacin increased the SGE amout of the LTR promotor. The opposite effect between T2EC and the LTR promotor system could have different origins:

- First, the LTR promotor is the only DNA region analyzed in the original study [37]. In our study, we analyzed the SGE variation of 92 genes previously selected to be relevant for avian erythropoiesis.
- Secondly, we analyzed the effect of Artemisinin and Indomethacin on SGE in T2EC as a sum of each effect on numerous genes. For some of them, the level of SGE was increased while for the others, the level of SGE was decreased.
- Finally, the cells used in the original study and ours are completely different. The original study used a line of human T-lymphocytes whereas we used a primary culture of avian erythroid progenitors.

MB-3 is an inhibitor of GCN5, a histone acetyl transferase (HAT) that activates global gene expression [60]. Even in such a seemingly well-defined case, it should nevertheless be remembered that a very complex relationship may lie between the biochemical action of a drug (HAT inhibition) and its biological effect on SGE [19].

Considering these different functions, it is hard to imagine that all these drugs have in common anything else than their ability to modulate SGE in T2EC. Nevertheless, it is important to note that it remains difficult to be certain that the effect of the drugs on differentiation is due to a direct effect of the drug on its know target or on some off-target effect. One method to resolve this issue would be to act on the pathway that the drug targets, without using the molecule (*e.g*. knockdown a drug’s target). For MB-3, a knockdown of *KAT2A* was performed in parallel of the use of MB-3 in mesendodermal differentiation (Moris et al., 2018). Both affected differentiation and the SGE in the same manner, demonstrating that the results indeed seem to be directly related to the biological inhibition of *KAT2A* and not to another independent pathway. Performing the same experiments for the two other drugs would clearly address this issue, although this might be more challenging for Artemisinin, for which many targets have been identified [56].

The question then arises of the mechanisms through which these different drugs modulate SGE. We first assessed whether these drugs affected the entropy of the same genes. For Indomethacin and Artemisinin, we showed that indeed the entropy of some of the same genes were affected but with a weak correlation. In contrast, MB-3 increased SGE through a different set of genes. This tends to indicate that the modulation of cell-to-cell variability *per se*, relatively independently of the gene function involved, is related to a modification of the differentiation process (see below).

Even if the correlation is weak, drugs reducing cell-to-cell gene expression variability seemed to affect the entropy of the same genes. Indeed, Artemisinin and Indomethacin both increased the cell-to-cell variability of 4 genes: *ALAS1*, *TPP1*, *MFSD2B* and *MKNK2*. *ALAS1* gene encodes for a protein involved in the heme production in red blood cells [61]. *TPP1* gene, previously named *CLN2*, encodes for a soluble lysosomal enzyme involved in metabolism [62]. MFSD2B is a lipid transporter released by erythrocytes and important in bone homeostasis [63, 64]. *MKNK2* encodes the protein MNK2, which is a downstream kinase activated by MAPK1 [65]. All these genes are related to erythocytes but no common function emerges, which prohibits the identification of a core network gene, targeted by drugs to reduce simultaneously the level of SGE and the differentiation. Moreover, Artemisinin and Indomethacin decreased the noise of *LDHA* and *MTFR1* genes, whereas MB-3 increased it. These genes are known to be involved in the metabolic switch that has been shown to be a key for the avian erythroid differentiation process [31]. Drugs could control SGE and differentiation though the modulation of the metabolic pathway needed to progress during the erythropoiesis. For further analyses, it could be interesting to further investigate these metabolic genes and the influence of their SGE change on the differentiation process.

It is important to note that our work focuses on genes that encode for erythroid differentiation and might not represent all genes. Overall it is advisable to use more caution when interpreting the importance of the role of gene affected by drugs in this study.

Thus, we can not exclude that there may exist another set of genes preferentially affected by drug that increase SGE.

We then investigated a potential role for variation in the mean gene expression that could explain the variation of the level of SGE.

Modifying the level of SGE is accompanied by a variation in the mean gene expression level for two drugs out of three. The decrease of mean gene expression under MB-3 treatment has been shown not to be significant in a different system [13]. Also, it has not been reported that Artemisinin affects mean gene expression in any other cellular system. However, the fact that Indomethacin treatment decreased gene-wise entropy clearly without affecting the mean gene-wise expression level reinforces the fact that the modification on the differentiation process is not associated to a modification in mean gene expression but only to a non-specific modulation of SGE.

Collectively, these results suggest that neither common genes nor common mechanisms could explain the observed effect of the three drugs simultaneously. This reinforces the fact that modulation of cell-to-cell variability is strongly accompanied by a change of differentiation, independently of a gene function or a specific mechanism involved.

This could be explained by adopting a dynamical systems view on the differentiation process, in the wake of Waddington’s proposal [66]. In such a view, we could consider that in the highly dimensional gene expression space, an equilibrium cell state could be compared to a valley in an epigenetic landscape [26]. It has been shown that entropy is a useful tool for analyzing stochastic processes [67] and distinguishes between equilibrium and transition states [50]. When we reduce SGE using Indomethacin or Artemisinin, we dig the valley, limiting the ability of cells to escape from a self-renewal equilibrium. Their probability to reach the new equilibrium state is reduced. Inversely, when we increase SGE using MB-3, we flatten the valley and improve the ability of cells to explore a larger dynamical landscape, and increase their probability to reach the new differentiated equilibrium state. Alternatively, we could think that drugs modulate differentiation dynamics, flattening or digging valleys, allowing cells to increase or decrease their probability to escape from the valley. Cells will experience a modulation of the amount of SGE as the consequence of their stability in the high dimensional gene expression space. Once cells achieve their journey, they stabilize their new gene expression pattern (the differentiated genetic profile) and return to a basal level of SGE [25, 26, 28]. In such a view, stochastic gene expression favours cells making the decision to differentiate, modifying the structure of the valley in which cells are moving. In a recent perspective, this same process of actively shaping the Waddington Landscape has been described in terms of a Plinko board, whose nail configuration, composition, and patterning can be modified towards forward stochastic design [12]. Similarly to our initial description [31], the variation of cell-to-cell gene expression in other differentiation systems has been recently described [13, 32–34, 68]. Furthermore, a strong evidence of the relation between transcriptional heterogeneity and cell fate transitions was demonstrated recently through destabilization of the histone acetylation leading simultaneously to an increase of SGE and differentiation of mouse embryonic stem cells [13] and myogenic progenitors [36]. This is fully backed up by our own data that also establish that the inverse (reducing simultaneously differentiation and SGE) can also be demonstrated.

## Conclusion

We show in primary erythroid progenitor cells that a pharmacological modification of SGE is consistently accompanied by a modulation of the differentiation process. Similar experiments using the design principles described above can be used to help establish the contribution of variability to biological processes and to separate cause from consequence [45]. It could therefore be important to study the potential use of such drugs in differentiation-related diseases such as tumoral cell progression [69], as exemplified by chronic myeloid leukemia [12, 70, 71], paving the way to a “treatment by noise” of at least some cancer-related diseases.

## Supporting information

S1 Fig *In sillico* interaction analysis between drugs’ targets. Representation of connections among known three drugs’ targets using the STRING database (http://string.embl.de/). Each edge between two proteins corresponds to a known association between those proteins. In this figure, the two cyclooxygenases COX-1 and COX-2 are respectively named PTGS1 and PTGS2 for prostaglandin-endoperoxide synthase (their official name).
(PDF)

S2 Fig Gene expression distributions. For each treatment, we represent the gene expression distribution for the genes with the most negatively affected entropy (left panel) and for the genes with the most positively affected entropy (right panel), as defined in Table 1. We display the distribution for the treated condition in red and the distribution for the control condition in black.
(PDF)

S3 Fig The model reproduces the cellular kinetics observed *in vitro*. Simulation of the model in the untreated (black) and treated cases (color). Solid lines represent a simulation of the best model selected by Akaike’s weights. Dots and triangles are the experimental data (n = 3). On the left and the center are respectively displayed the total number of living cells in self-renewing (LM1) and differentiated (DM17) media (in log-scale). On the right are displayed the fraction of differentiated cells (in percentage) in differentiated (DM17) medium.
(PDF)

S4 Fig Drugs do not change the erythroid commitment. T2EC were induced to differentiate for 24 (solid lines) and 48 (dashed lines) hours and subsequently seeded back in self-renewal conditions. Cells were then counted every day for 3 days. The data shown are the mean ± standard deviation calculated on the basis of three independent experiments. The growth ratio was computed as the cell number divided by the total cells at day 0.
(PDF)

S5 Fig Model selection by Akaike’s weights. Shown are the Akaike weights of the models, sorted from best to worst. For readability, the worst models were omitted. For each drug, the coloured bars represent the models which amount to 95% of the overall Akaike’s weight.
(PDF)

S6 Fig Drug toxicity in T2EC. Measurements of drug toxicity in self-renewal medium (left panel) and in differentiation medium (right panel) have been performed. In black are represented the control conditions. Treated conditions are represented in color. Cell toxicity for Indomethacin, Artemisinin treatment and their control was performed at 48h of differentiation. For MB-3 treatment and its control, the cell toxicity was performed at 24h of differentiation. Each drugs toxicity has been tested with the adequat concentration used in the study. Wilcoxon tests were performed between each pair of control and treated conditions. All tests were negative for a significant difference between control and treatment (p-value < 0.05, n = 3).
(PDF)
