# Supplementary figures and images for: Drugs modulating stochastic gene expression affect the erythroid differentiation process
Source: PLoS One. 2019 Nov 21;14(11):e0225166. doi: 10.1371/journal.pone.0225166 (PMC6872177; doi:10.1371/journal.pone.0225166)

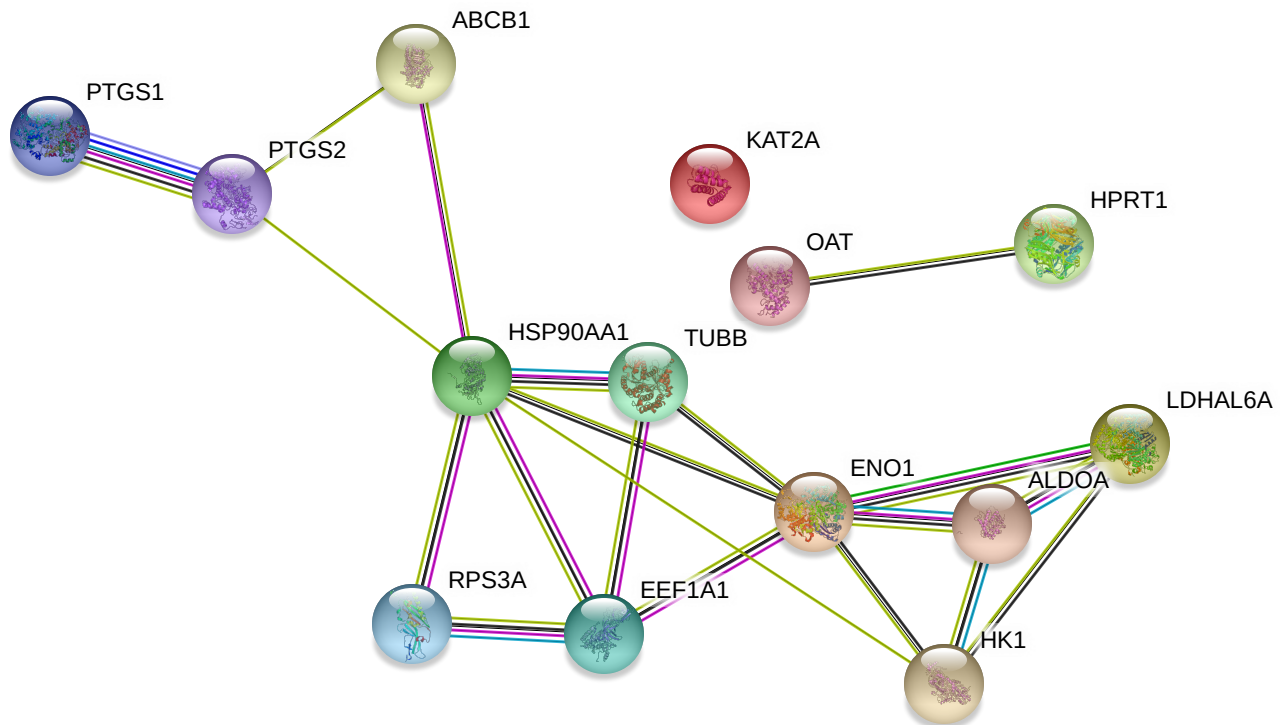

Supplement: S1 Fig — Representation of connections among known three drugs’ targets using the STRING database (http://string.embl.de/). Each edge between two proteins corresponds to a known association between those proteins. In this figure, the two cyclooxygenases COX-1 and COX-2 are respectively named PTGS1 and PTGS2 for prostaglandin-endoperoxide synthase (their official name). (PDF) [file pone.0225166.s001.pdf]

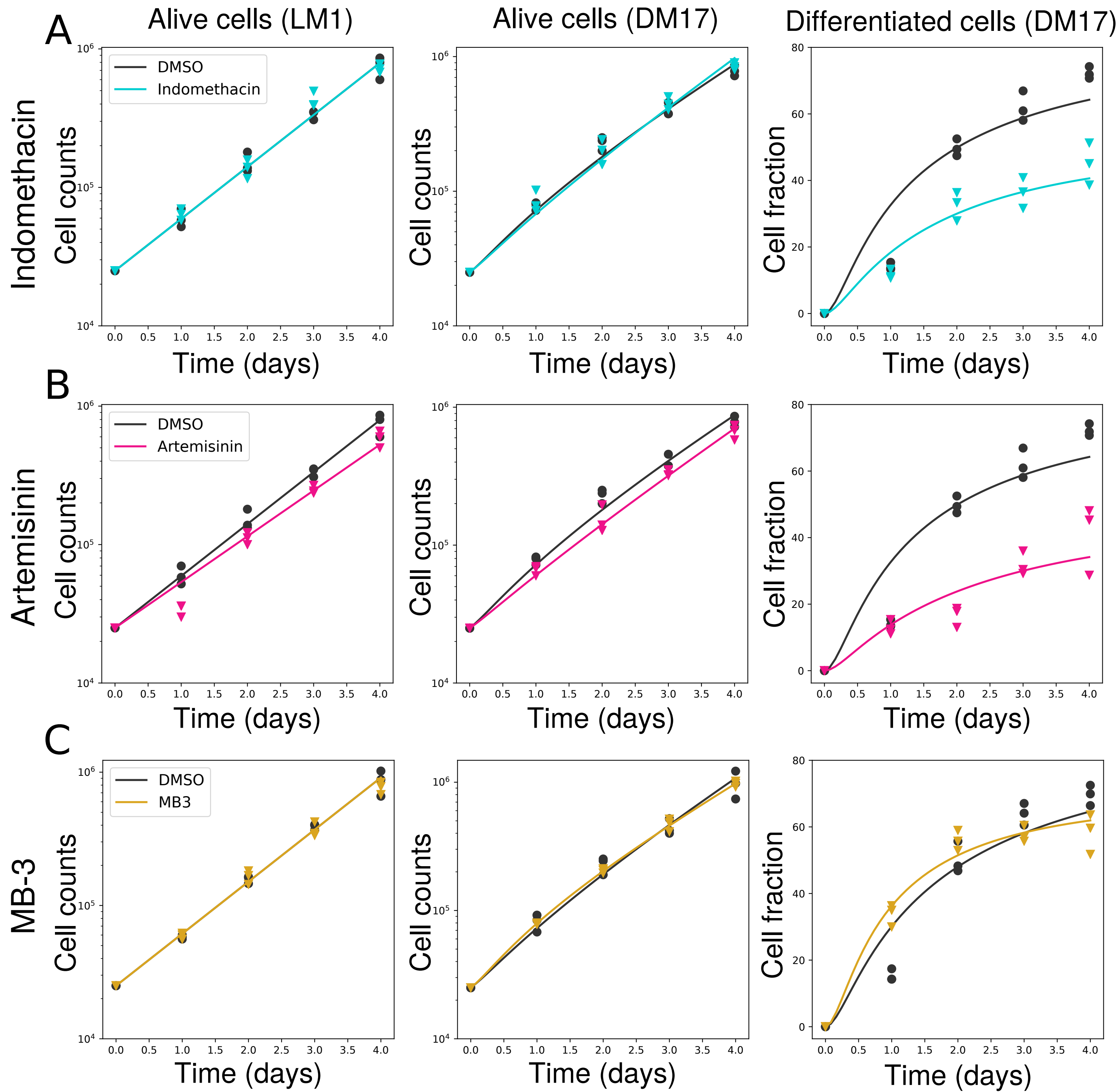

Supplement: S3 Fig — Simulation of the model in the untreated (black) and treated cases (color). Solid lines represent a simulation of the best model selected by Akaike’s weights. Dots and triangles are the experimental data (n = 3). On the left and the center are respectively displayed the total number of living cells in self-renewing (LM1) and differentiated (DM17) media (in log-scale). On the right are displayed the fraction of differentiated cells (in percentage) in differentiated (DM17) medium. (PDF) [file pone.0225166.s003.pdf]

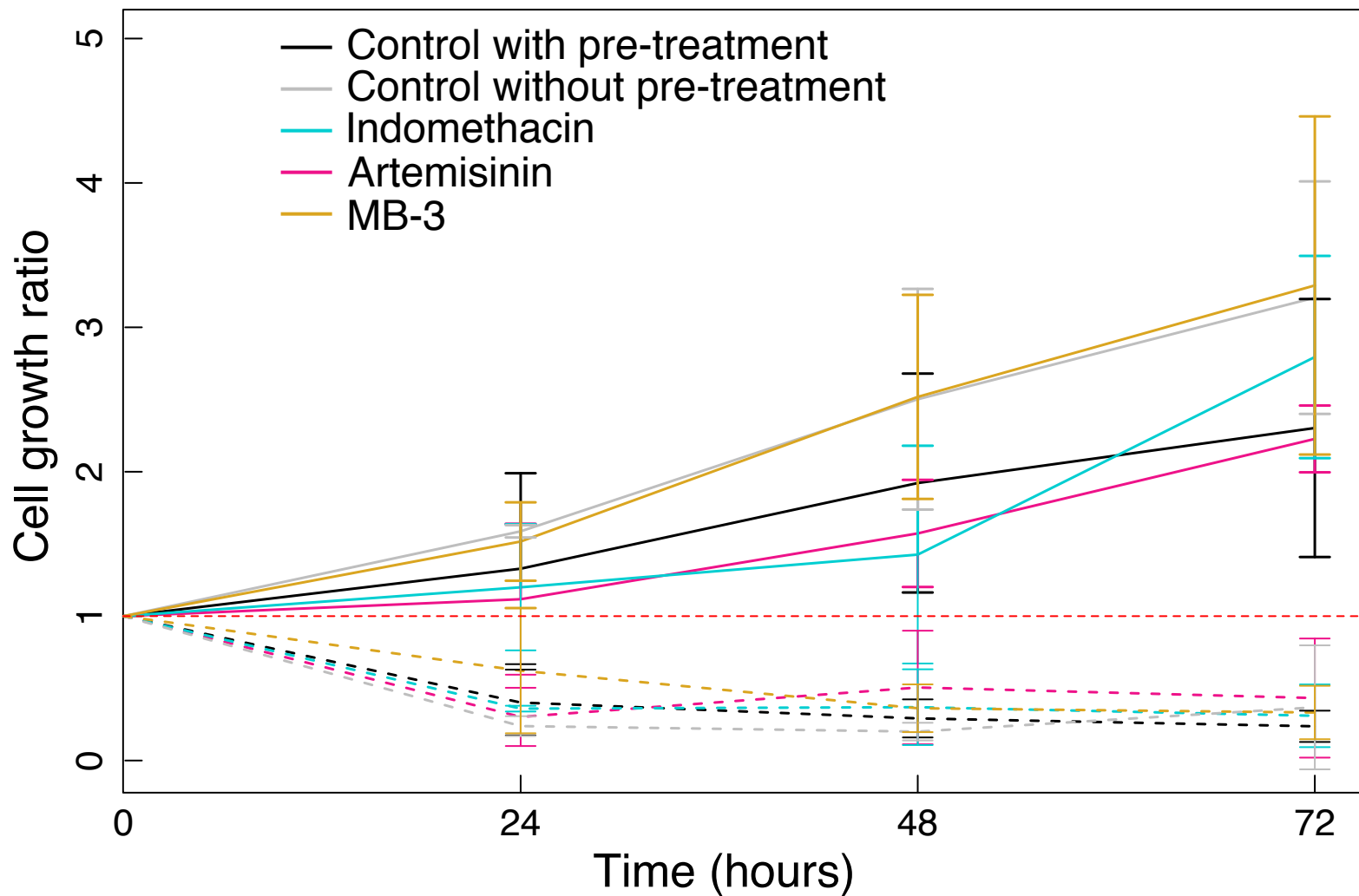

Supplement: S4 Fig — T2EC were induced to differentiate for 24 (solid lines) and 48 (dashed lines) hours and subsequently seeded back in self-renewal conditions. Cells were then counted every day for 3 days. The data shown are the mean ± standard deviation calculated on the basis of three independent experiments. The growth ratio was computed as the cell number divided by the total cells at day 0. (PDF) [file pone.0225166.s004.pdf]

# Indomethacin

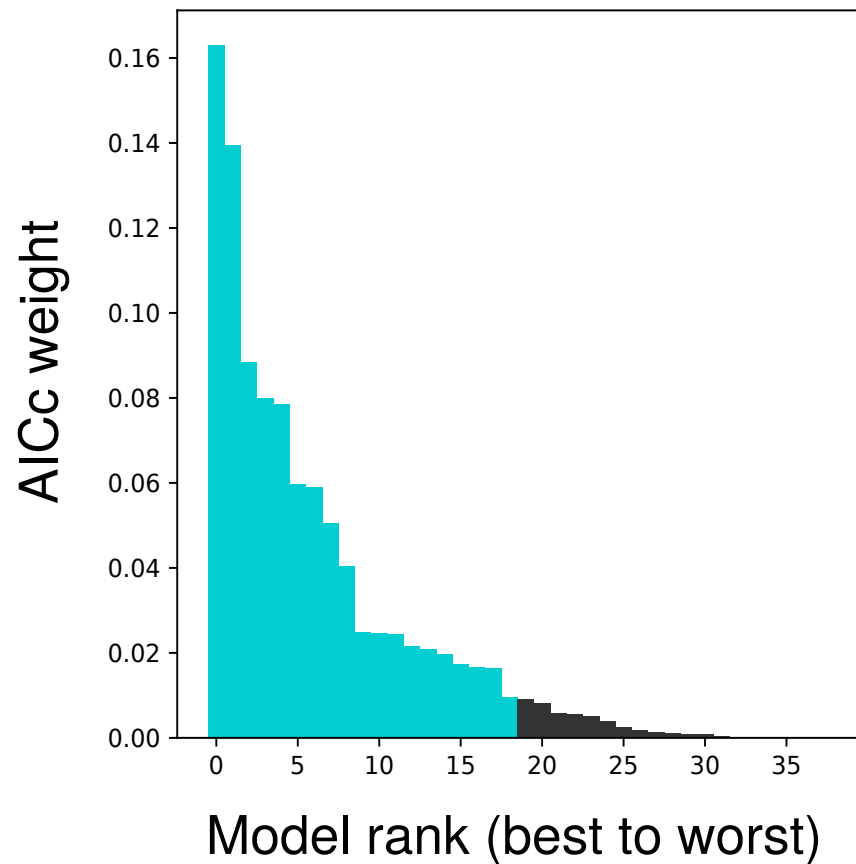

# Artemisinin

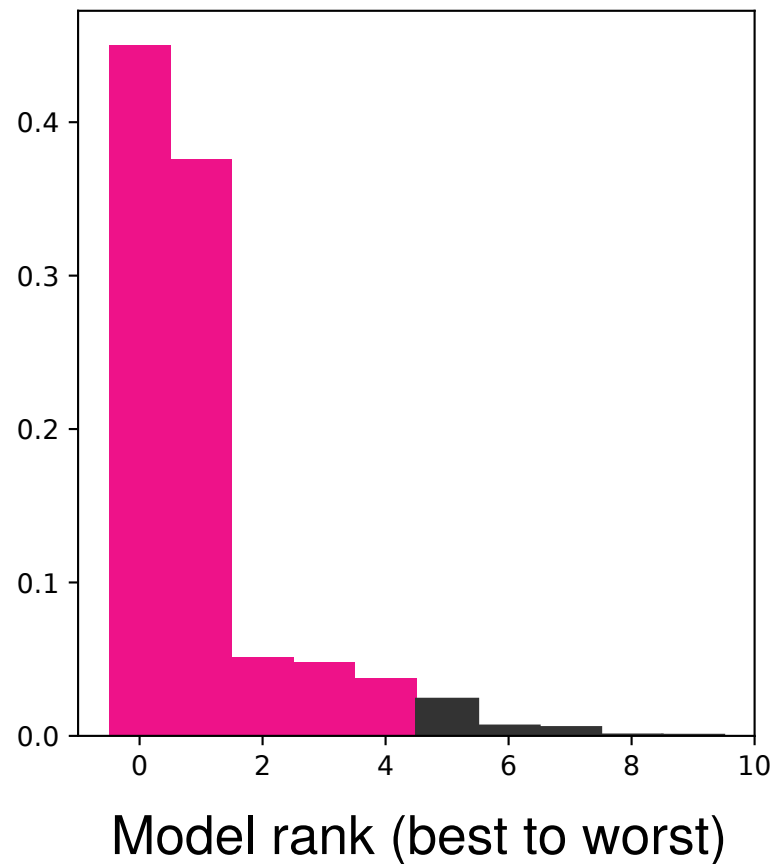

# MB-3

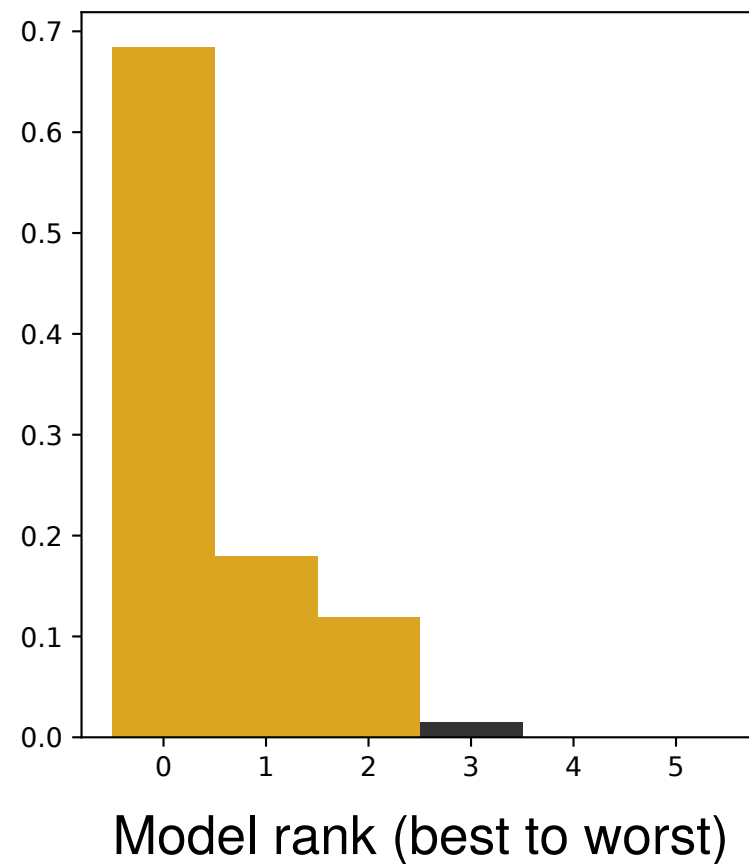

Supplement: S5 Fig — Shown are the Akaike weights of the models, sorted from best to worst. For readability, the worst models were omitted. For each drug, the coloured bars represent the models which amount to 95% of the overall Akaike’s weight. (PDF) [file pone.0225166.s005.pdf]

Self-renewal medium

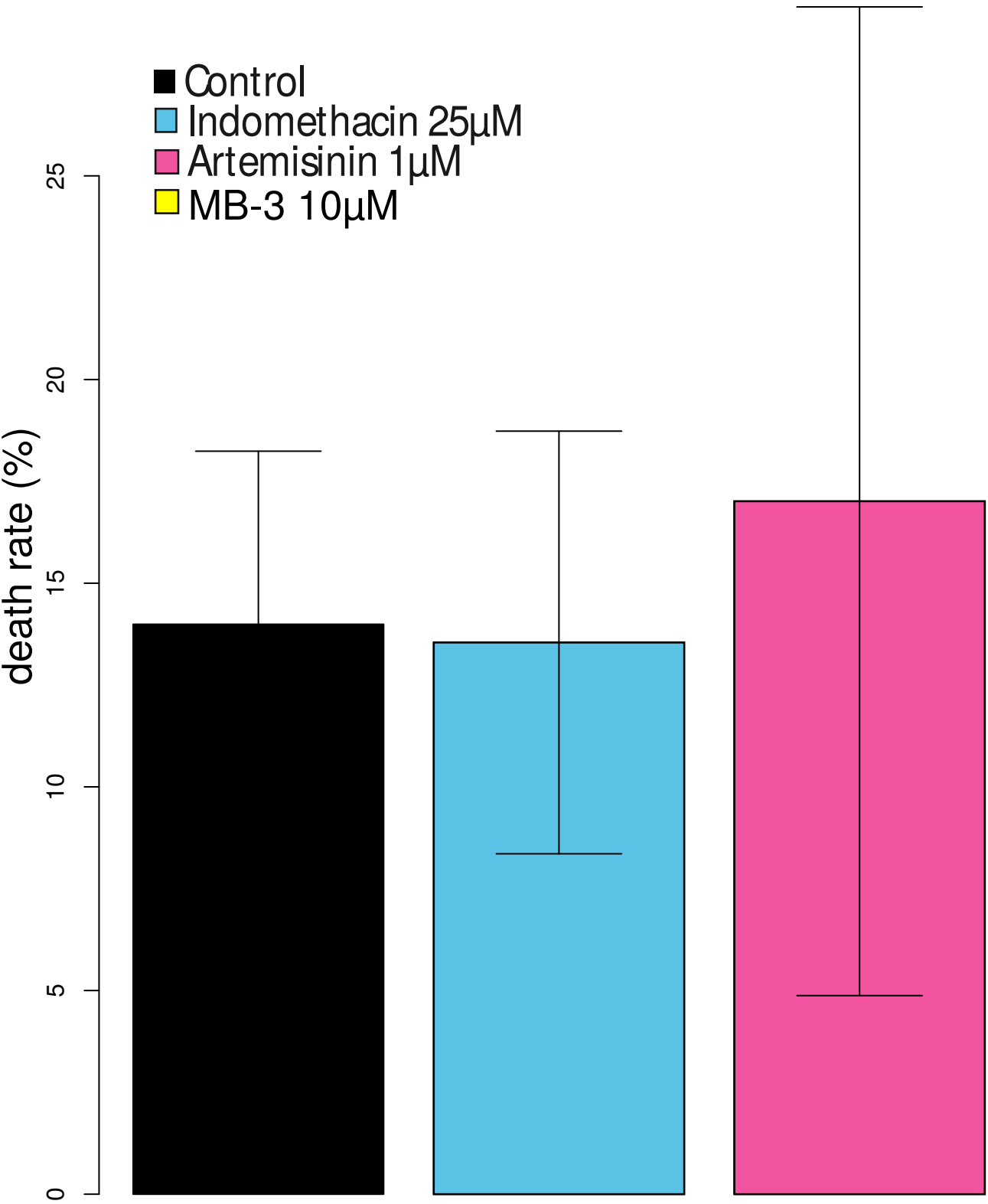

Differentiation medium

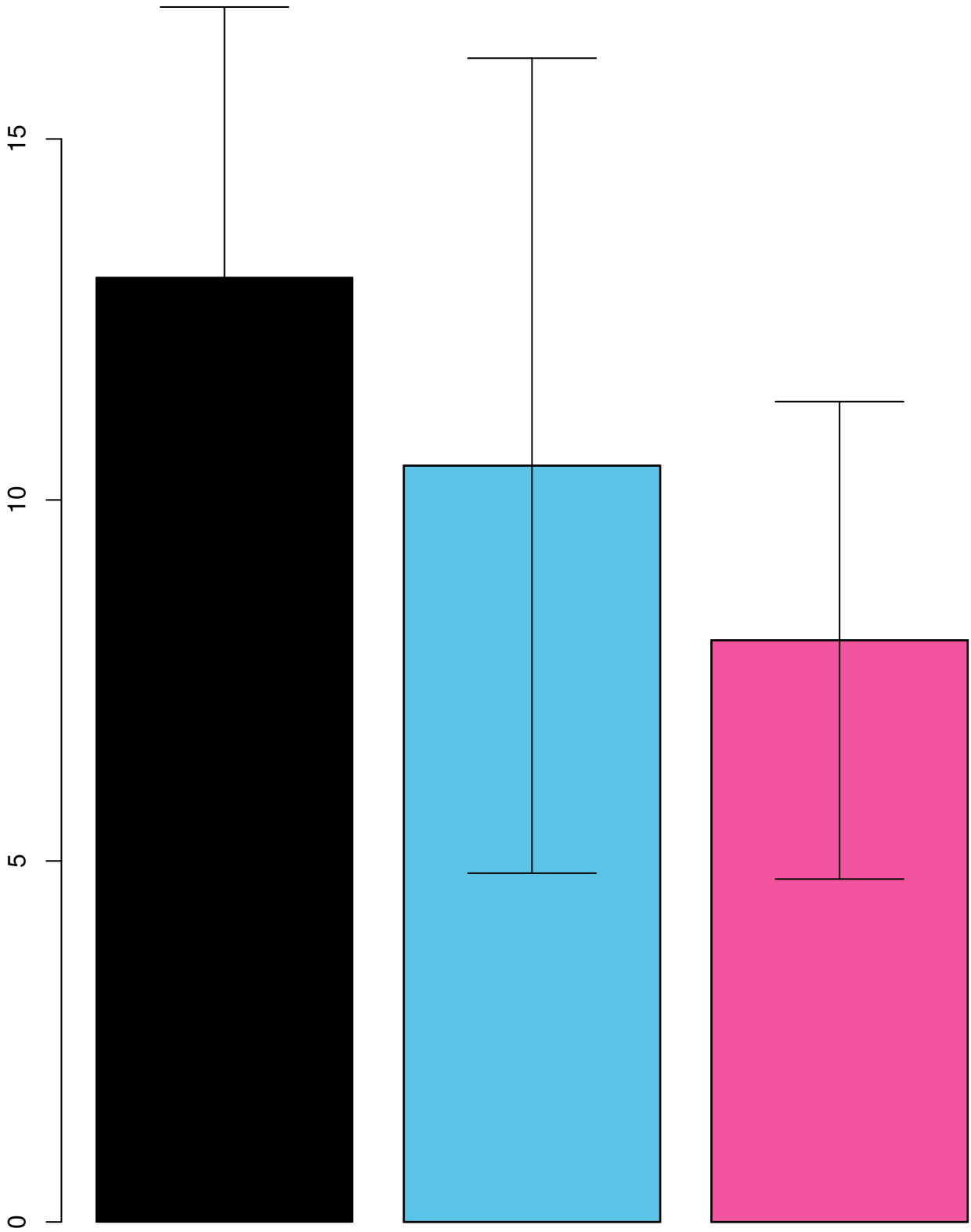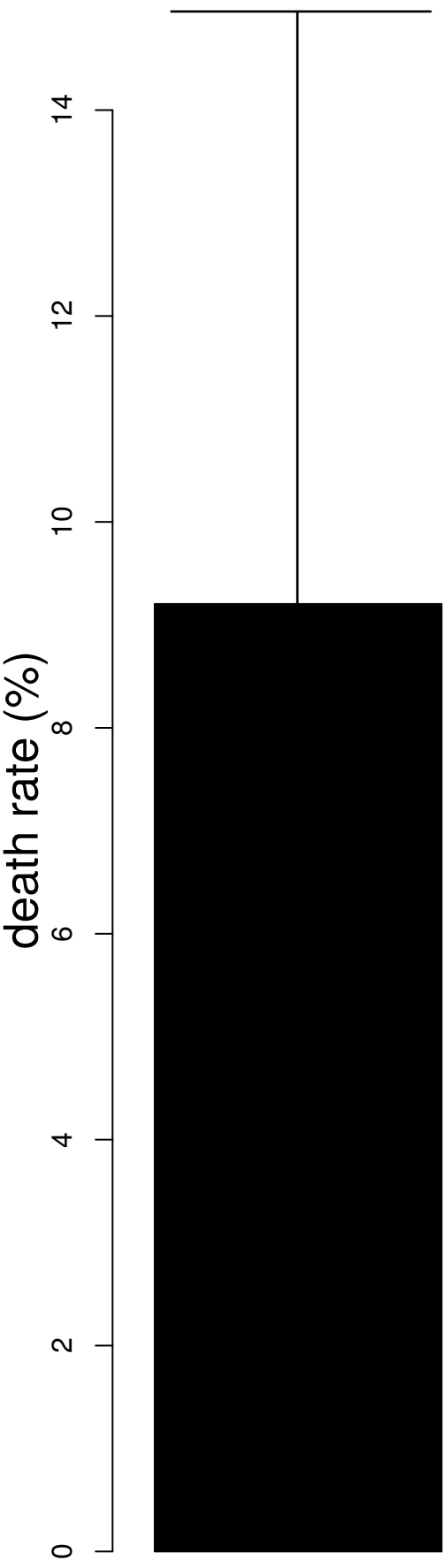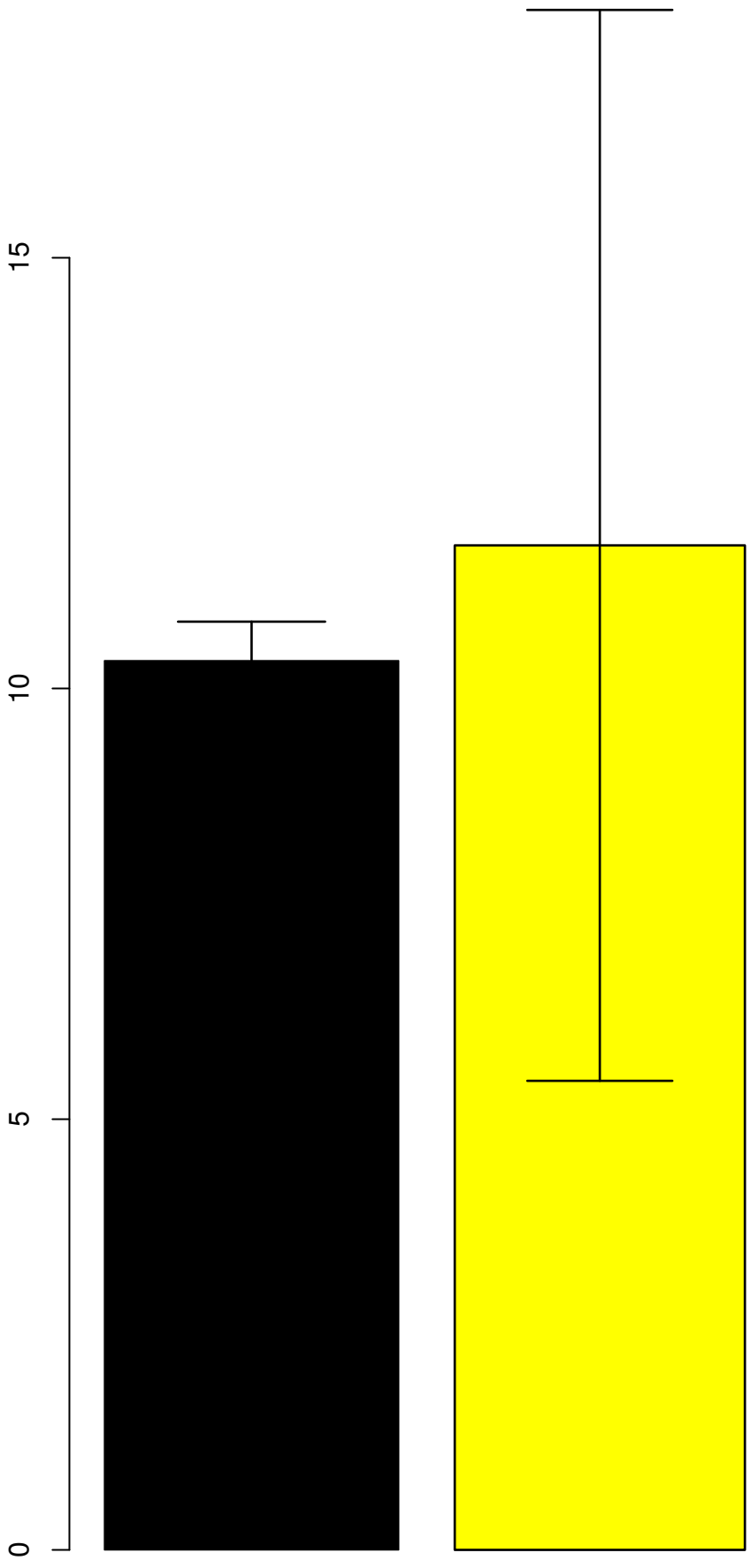

Supplement: S6 Fig — Measurements of drug toxicity in self-renewal medium (left panel) and in differentiation medium (right panel) have been performed. In black are represented the control conditions. Treated conditions are represented in color. Cell toxicity for Indomethacin, Artemisinin treatment and their control was performed at 48h of differentiation. For MB-3 treatment and its control, the cell toxicity was performed at 24h of differentiation. Each drugs toxicity has been tested with the adequat concentration used in the study. Wilcoxon tests were performed between each pair of control and treated conditions. All tests were negative for a significant difference between control and treatment (p-value < 0.05, n = 3). (PDF) [file pone.0225166.s006.pdf]
