# Supplementary material for: Drugs modulating stochastic gene expression affect the erythroid differentiation process
Source: PLoS One. 2019 Nov 21;14(11):e0225166. doi: 10.1371/journal.pone.0225166 (PMC6872177; doi:10.1371/journal.pone.0225166)
Supplement: S2 Fig — For each treatment, we represent the gene expression distribution for the genes with the most negatively affected entropy (left panel) and for the genes with the most positively affected entropy (right panel), as defined in Table 1. We display the distribution for the treated condition in red and the distribution for the control condition in black. (PDF) [file pone.0225166.s002.pdf]

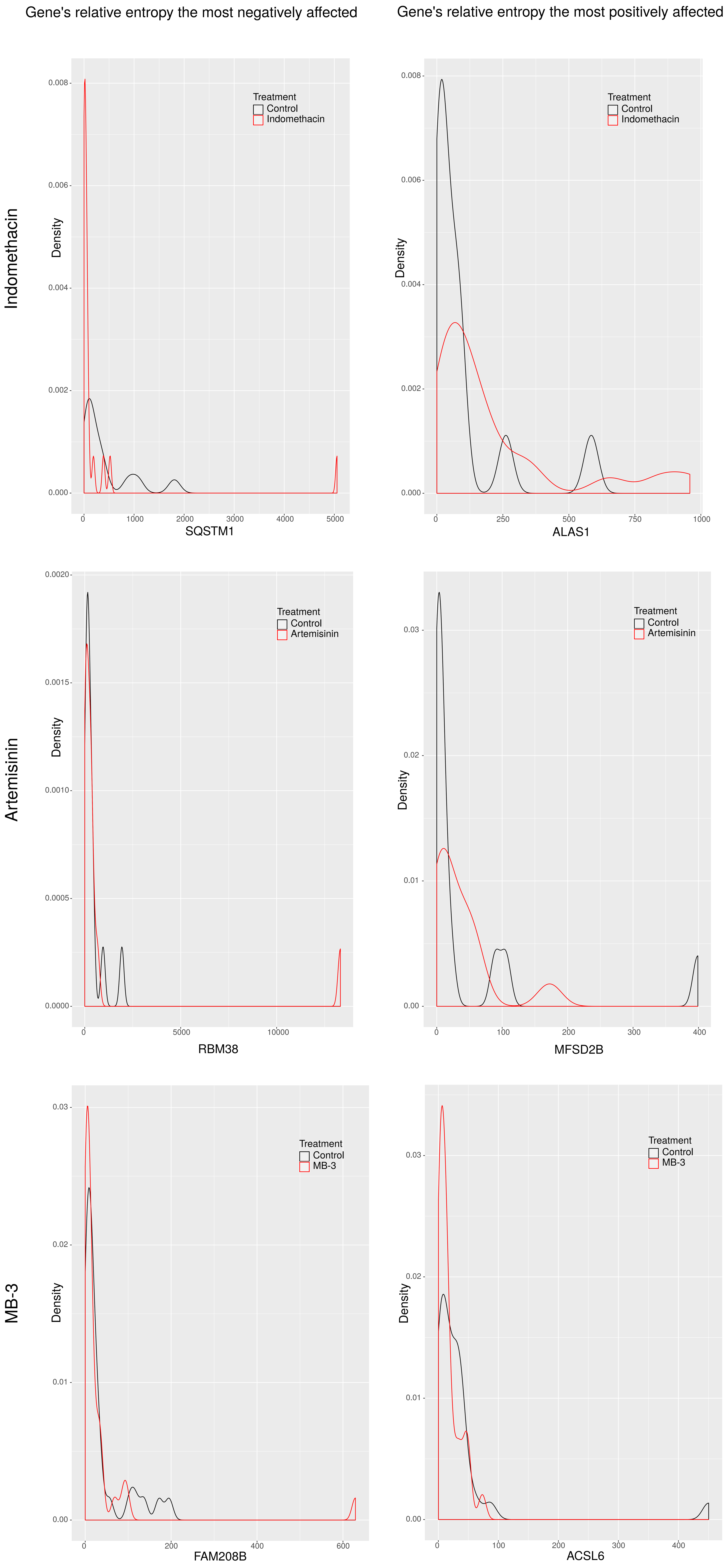
Gene's relative entropy the most negatively affected
Gene's relative entropy the most positively affected
Indomethacin
Artemisinin
MB-3
Density
Treatment
Control
Indomethacin
0.000
0.002
0.004
0.006
0.008
0
1000
2000
3000
4000
5000
SQSTM1
0
250
500
750
1000
ALAS1
Artemisinin
0.0000
0.0005
0.0010
0.0015
0.0020
5000
10000
RBM38
0.00
0.01
0.02
0.03
100
200
300
400
MFSD2B
MB-3
200
400
600
FAM208B
ACSL6
